# Effects of L-citrulline supplementation and watermelon intake on arterial stiffness and endothelial function in middle-aged and older adults: a systematic review and meta-analysis of randomized controlled trials

**DOI:** 10.3389/fnut.2025.1632952

**Published:** 2025-11-13

**Authors:** Ping Luo, Ziyi Li, Kang Liu, Weifeng Gao

**Affiliations:** 1 School of Physical Education, Wuhan Sports University, Wuhan, China; 2 Physical Education and Sports, Belarusian State University of Physical Culture, Minsk, Belarus; 3 School of Physical Education and Health, Nanning Normal University, Nanning, China

**Keywords:** L-citrulline, watermelon, arterial stiffness, pulse wave velocity, flow-mediated dilation

## Abstract

**Objectives:**

To determine and explore the effects of L-citrulline supplementation and watermelon intake on arterial stiffness (AS) and endothelial function in middle-aged and elderly individuals.

**Participants:**

Middle-aged and elderly adults.

**Designs:**

A systematic review and meta-analysis of randomized controlled trials (RCTs).

**Methods:**

A comprehensive search was conducted across four major electronic databases (PubMed, Cochrane, EMBASE, and Web of Science), covering the period from database inception to May 1, 2025. The quality of included studies was assessed using the Cochrane Risk of Bias Assessment tool 2.0. Data analysis was performed with RevMan 5.4.1 software; pulse wave velocity (PWV) data were analyzed using a random-effects model to pool effect sizes, while flow-mediated dilation (FMD) data were analyzed using a fixed-effects model to pool effect sizes. Heterogeneity was evaluated using the chi-square-based Cochran's *Q* test (*p* < 0.10) and the *I*^2^ statistic.

**Results:**

This systematic review and meta-analysis included 8 RCTs with a total of 176 participants. The results showed that L-citrulline supplementation significantly improved FMD [1.81 (95% CI: 0.76 to 2.85), *p* = 0.0007]. Although L-citrulline supplementation did not significantly improve PWV [−0.14 (95% CI: −0.45 to 0.17), *p* = 0.37], a trend toward improvement was observed. Subgroup analyses indicated that L-citrulline supplementation had the most significant effect on ankle-brachial pulse wave velocity (BA-PWV) [−1.11 (95% CI: −1.37 to −0.85), *p* < 0.00001). However, watermelon supplementation did not significantly improve PWV and FMD in middle-aged and older adults.

**Conclusion:**

Supplementation with L-citrulline has a positive impact on vascular function in middle-aged and elderly individuals, significantly improving FMD. However, although there was no improvement in PWV, subgroup analysis results still show that L-citrulline supplementation significantly reduced BA-PWV. This suggests that this intervention may have potential application value in preventing and improving the risk of cardiovascular diseases (CVDs) in this population. However, watermelon intake did not significantly improve FMD and PWV in middle-aged and older adults, and there is insufficient relevant literature. Future large-scale studies are needed to confirm the effects of watermelon on vascular function.

**Systematic Review Registration:**

https://www.crd.york.ac.uk/prospero/CRD420251052954

## 1 Introduction

Cardiovascular diseases (CVDs) are the leading cause of mortality among non-communicable diseases, with a death rate twice that of other diseases (1). With the continuous growth of the elderly population, the prevalence of CVDs is also increasing (2, 3). Studies have shown that over 70% of adults have CVDs by the age of 70, with more than two-thirds of these patients also suffering from comorbid non-CVDs (4, 5). Arterial stiffness (AS) and endothelial dysfunction are common manifestations of vascular aging (6), which significantly increase the incidence of cardiovascular events and are among the primary causes of CVDs onset and progression (7). Therefore, effective interventions to improve AS and endothelial function in middle-aged and elderly populations are necessary to prevent or reduce the incidence of CVDs (2).

L-citrulline is a non-essential amino acid that can be obtained from fresh foods, particularly watermelon, and is commonly used as a dietary supplement (8–10). Existing research demonstrates that L-citrulline exerts a beneficial effect on vascular function in humans (11, 12). L-citrulline can be converted to L-arginine in the kidneys through the urea cycle (13–16). L-arginine serves as a substrate for endothelial nitric oxide synthase (eNOS) in endothelial cells to produce nitric oxide (NO) (17, 18). The generated NO acts as a signaling molecule that diffuses into vascular smooth muscle cells, inducing relaxation and resulting in vasodilation (18–20). **Current experimental studies indicate that L-citrulline supplementation and watermelon intake may have a positive impact on (AS and endothelial function in middle-aged and elderly populations. However, the results of these studies still exhibit some discrepancies, and a unified conclusion has yet to be established. For example, Ochiai et al. conducted a 7-day L-citrulline supplementation study in middle-aged men, which suggested that oral L-citrulline supplementation may functionally improve AS without relying on changes in blood pressure** **(****21****). In contrast, Ellis et al. performed a four-week supplementation of 100% watermelon juice in postmenopausal women, finding no improvement in vascular function in that population** **(****22****). Additionally, there currently lacks dedicated quantitative analyses specifically assessing the effects of L-citrulline or watermelon supplementation on vascular function in older adults**.

Given that middle-aged and elderly populations commonly face risks of vascular function decline, and existing studies have shown that L-citrulline or watermelon supplementation positively affects vascular function, this study will select pulse wave velocity (PWV) and flow-mediated dilation (FMD)—two gold-standard indicators for assessing AS and endothelial function—as the primary evaluation metrics. The aim is to systematically assess the effects of L-citrulline and watermelon supplementation on AS and endothelial function in middle-aged and elderly individuals.

## 2 Materials and methods

### 2.1 Trial registration

This systematic review and meta-analysis of randomized controlled trials (RCTs) is reported strictly in accordance with the PRISMA (Preferred Reporting Items for Systematic Reviews and Meta-Analyses) guidelines and has been registered on the PROSPERO platform (23). The registration number is as follows: CRD420251052954.

### 2.2 Search strategy

A comprehensive search was conducted across four major electronic databases (PubMed, Cochrane, Embase, and Web of Science), covering the period from database inception to May 1, 2025. To capture relevant free-text terms, we performed subject heading searches on the keywords Citrulline, Citrullus, and Vascular Stiffness in the databases. The specific search strategy is as follows:

(“citrulline” OR “citrulline malate” OR “l-citrulline” OR “citrullus” OR “watermelon” OR “citrullus lanatus”) AND (“vascular stiffness” OR “arterial stiffness” OR “aortic stiffness” OR “pulse wave velocity” OR “PWV” OR “endothelial function” OR “endothelium” OR “vascular endothelium” OR “vascular reactivity” OR “vascular” OR “vasodilation” OR “brachial artery” OR “brachial artery dilation” OR “flow mediated dilation” OR “artery blood flow” OR “artery dilation” OR “flow mediated” OR “FMD”) AND (“random” OR “randomized controlled trial” OR “RCT”). In addition, relevant reviews were read and examined, and literature published after May 1, 2025, was traced to ensure the comprehensiveness of the included studies. The detailed search strategies and records are provided in Supplementary material 1.

The search strategy used in PubMed is as follows:

Set1:(“Citrulline”[Mesh]) OR ((citrulline malate[Title/Abstract]) OR (l-citrulline[Title/Abstract]))

Set2:(“Citrullus”[Mesh]) OR ((watermelon[Title/Abstract]) OR (citrullus lanatus[Title/Abstract]))

Set3:(“Vascular Stiffness”[Mesh]) OR ((((arterial stiffness[Title/Abstract]) OR (aortic stiffness[Title/Abstract])) OR (pulse wave velocity[Title/Abstract])) OR (PWV[Title/Abstract]))

Set4:((((((((((((endothelial function[Title/Abstract]) OR (endothelium[Title/Abstract])) OR (vascular endothelium[Title/Abstract])) OR (vascular reactivity[Title/Abstract])) OR (vascular[Title/Abstract])) OR (vasodilation[Title/Abstract])) OR (brachial artery[Title/Abstract])) OR (brachial artery dilation[Title/Abstract])) OR (flow mediated dilation[Title/Abstract])) OR (artery blood flow[Title/Abstract])) OR (artery dilation[Title/Abstract])) OR (flow mediated[Title/Abstract])) OR (FMD[Title/Abstract])

Set5: ((random[Title/Abstract]) OR (randomized controlled trial[Title/Abstract])) OR (RCT[Title/Abstract])

Set6: #1 OR #2

Set7: #3 OR #4

Set8: #5 AND #6 AND #7

### 2.3 Eligibility criteria

Studies will be included in this meta-analysis if they meet all of the following criteria: (1) subjects are middle-aged and older adults (aged ≥45 years); (2) the intervention group receives L-citrulline supplementation or watermelon intake (including combinations with other forms of supplementation); (3) **the control group receives a placebo;** (4) outcome measures include pre- and post-intervention PWV data for any arterial segment or pre- and post-intervention FMD data; (5) the study design is an RCT.

Studies will be excluded if they meet any of the following criteria: (1) non-English publications; (2) duplicate publications; (3) **Based on the existing literature, articles with supplementation periods of less than 1 day were excluded** **(****24****)**.

### 2.4 Risk of bias and quality assessment of the literature

Risk of bias assessment for the included studies was conducted using the Cochrane Risk of Bias tool (RoB 2) (25). This tool comprises five distinct domains: (1) randomisation process; (2) deviations from the intended interventions; (3) missing outcome data; (4) measurement of the outcome; (5) selection of the reported result.

The modified JADAD scale was used to assess the quality of the included studies (26). The modified JADAD scoring evaluates random sequence generation, allocation concealment, blinding, and whether details of participant withdrawals or dropouts are described. A total score of 4–7 indicates high-quality studies, while a score of 1–3 indicates low-quality studies.

To ensure the reliability of the risk of bias and quality assessment process and to minimize subjective bias, the evaluations were independently conducted and cross-checked by two researchers (PL and ZYL). Any disagreements arising during the screening process were resolved through discussion, and if discrepancies persisted, a third researcher (WFG) made the final decision.

### 2.5 Data extraction

Two experienced researchers (PL and ZYL) independently extracted the required data using the same Excel spreadsheet. In case of any discrepancies, a third researcher (WFG) intervened and facilitated discussion until consensus was reached. The following key information was primarily extracted: (1) basic study information (first author, year of publication, and country); (2) participant information (population characteristics, gender ratio, and age); (3) intervention details (specific supplement administered, dosage, daily frequency, and duration of intervention); (4) outcome measures (PWV and FMD).

For the data used in statistical analysis, extraction was performed according to the following steps. First, the mean values and standard deviations (SD) of the outcome measures before and after the intervention were collected. The specific change was then calculated by subtracting the baseline mean from the post-intervention mean. The specific formula is as follows (27): MDdiff = Mpost – Mpre, where Mpre and Mpost represent the mean values of the outcome measure at baseline and after the intervention, respectively. The SD conversion formula is as follows (28): SDchange = $\sqrt{{\text{(SD}}_{\text{pre}}^{\text{2}}{\text{+ SD}}_{\text{post}}^{\text{2}}\text{) - (2}\times \text{Corr}\times \text{S}{\text{D}}_{\text{pre}}\;\times \text{S}{\text{D}}_{\text{post}}\text{)}}$, with the correlation coefficient (Corr) set at 0.5 according to the Cochrane Handbook guidelines. If essential data were missing, the original authors were contacted via email to obtain the necessary information.

### 2.6 Statistic analysis

This study primarily conducted statistical analysis on the obtained PWV and FMD data, using weighted mean difference (WMD) and its 95% confidence interval (CI) to evaluate the overall intervention effect. All statistical analyses were performed using RevMan 5.4.1 software (The Cochrane Collaboration, Copenhagen, Denmark, 2019). Forest plots were generated, and a p-value less than 0.05 was considered to indicate statistical significance. In this meta-analysis, the degree of heterogeneity among the included studies was first systematically assessed using Cochran's *Q* test and the *I*^2^ statistic. Specifically, the *I*^2^ value was used to quantify the magnitude of heterogeneity, corresponding to low (25%), moderate (50%), and high (75%) levels. When the *I*^2^ value exceeded 50%, indicating substantial heterogeneity, a random-effects model was applied to combine the data in order to better account for variability among studies. Conversely, when the *I*^2^ value was 50% or below, indicating low heterogeneity, a fixed-effects model was used to enhance the precision and statistical power of the estimates. To ensure the robustness of the results, sensitivity analyses were further conducted by sequentially excluding individual studies and observing changes in the overall effect size. This approach assessed the influence of each study on the combined results and evaluated the stability of the findings. Additionally, the possibility of publication bias was assessed using funnel plots and Egger's regression test. Funnel plots were generated with Stata 17.0 software (Stata Corporation, College Station, TX, USA) to visually inspect small-study effects and bias distribution. Egger's test evaluated the statistical significance of bias through the *p*-value; a *p*-value < 0.05 indicated significant publication bias, suggesting that the results should be interpreted with caution (29, 30).

## 3 Results

### 3.1 Study selection

**Figure 1:**
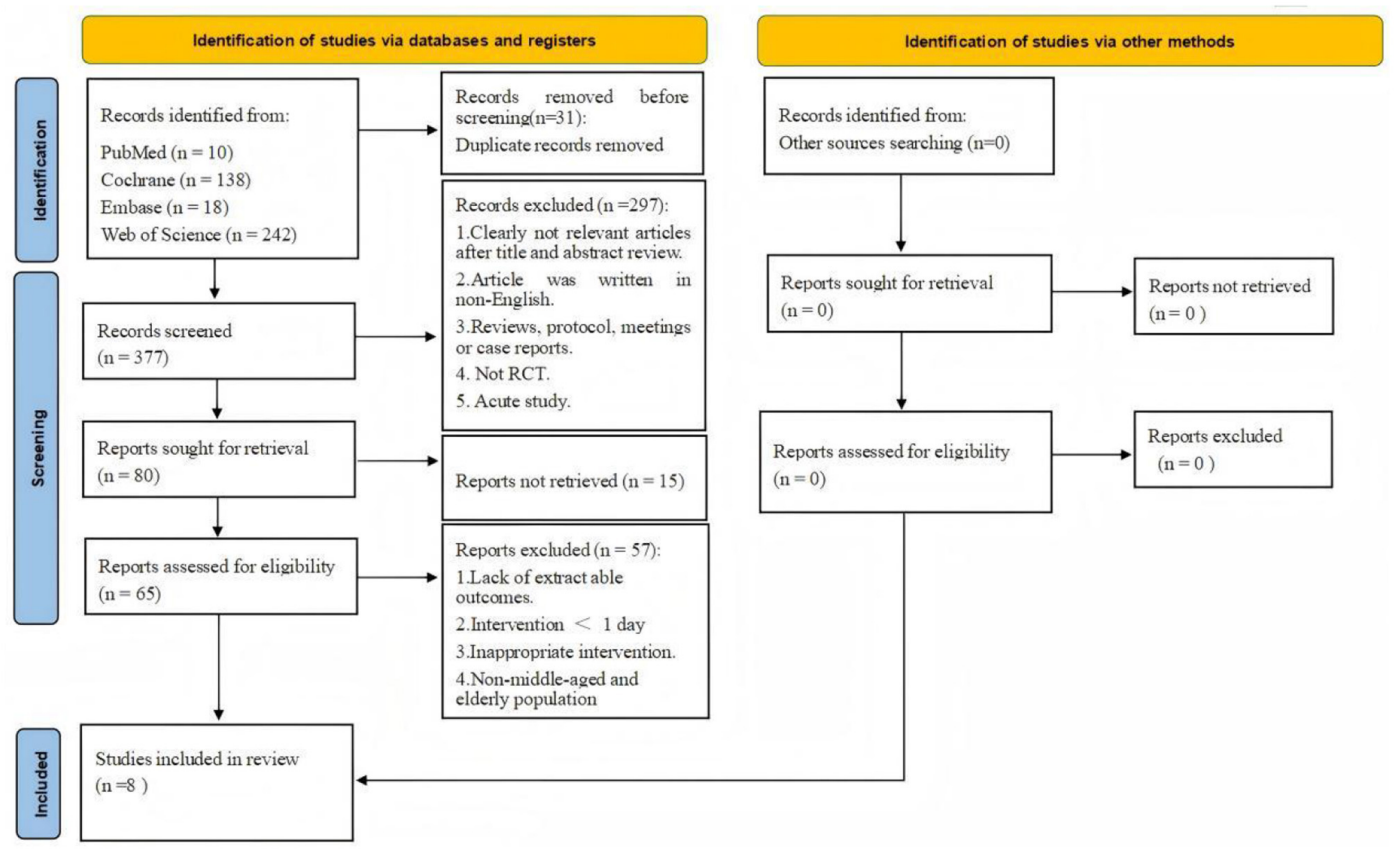
Flow diagram of systematic literature article search.

A comprehensive search of four major databases retrieved 408 articles, including 10 from PubMed, 138 from Cochrane, 18 from Embase, and 242 from Web of Science. After full-text screening, 8 RCTs investigating the effects of L-citrulline or watermelon supplementation on AS and endothelial function in middle-aged and elderly individuals were included (21, 22, 31–36). The detailed screening process is summarized in Figure 1, and the search records are provided in Supplementary Material 1.

### 3.2 Characteristics of included studies

As shown in Table 1, this meta-analysis included 8 RCTs investigating the effects of L-citrulline or watermelon supplementation on AS and vascular function in middle-aged and elderly individuals. These 8 studies involved a total of 176 participants, with 99 in the experimental groups and 77 in the control groups. Seven articles are from the United States (22, 31–36), and one article is from Japan (21). The ages of the participants range from 54 to 72 years. Four articles only included female participants (21, 22, 34, 36), one article only included male participants (32), and three articles included both male and female participants (31, 33, 35). Four articles focused on postmenopausal women (22, 32, 34, 36), among which one article included participants with hypertension (31). One article involved participants from the hypertensive population (34). All participants in the articles were middle-aged and older adults.

**Table 1:** Characteristics of included studies.

**First author, year**	**Country**	**Population**	**Sample size (EG/CG)**	**Proportion of females (%)**	**Age (EG/CG)**	**Supplement (EG)**	**Daily dosage frequency**	**Daily dose**	**Intervention period (Week)**	**Outcome measures**
Ochiai, 2010	Japan	Middle-aged men	8 7	100	58.5 ± 5.0 58 ± 3.9	L-citrulline	1	5.6G	1	BA-PWV
Figueroa, 2012	America	Pre-hypertensive population	5 4	55	54 ± 3	L-citrulline + L-arginine	2	2.1G + 1.3G	6	CF-PWV
Figueroa, 2013	America	Post menopausal women	6 6	0	57 ± 1	L-citrulline + L-arginine	3	4G + 2G	6	BA-PWV
Gonzales, 2017	America	Alder adults	13 12	52	70 ± 5	L-citrulline	2	6G	2	CF-PWV
Ellis, 2021	America	Postmenopausal women	11 8	100	60 ± 4.30	Watermelon juice	2	360 ML	4	PWV, FMD
Maharaj, 2022	America	Hypertensive Postmenopausal women	14 11	100	61 ± 6 64 ± 6	L-citrulline	2	10G	4	CF-PWV, FMD
Jaime, 2022	America	Alder adults	16 16	62	72.5 ± 7.3	L-citrulline	2	6G	6	CF-PWV
Figueroa, 2023	America	Postmenopausal women	26 13	100	58 ± 4 60 ± 5	L-citrulline L-citrulline + Glutathione	2	10G 2G + 200MG	4	CF-PWV, CR-PWV, CD-PWV, FA-WPV, FMD

EG, experimental group; CG, control group; G, gram; ML, milliliter; MG, milligram; NR, not reported; CF, carotid-femoral; BA, brachial-ankle; CR, carotid-radial; CD, carotid-distal; FA, femoral-ankle; PWV, pulse wave velocity; FMD, flow-mediated dilation.

In terms of intervention, all studies were randomized controlled trials, with seven articles implementing a double-blind intervention (21, 22, 31, 33–36), while one study did not report on the double-blind method (32). Five studies involved only L-citrulline supplementation (21, 33–36), two studies combined L-citrulline with L-arginine supplementation (31, 32), one article focused on watermelon juice supplementation (22), and one article involved L-citrulline combined with glutathione supplementation (36). Six articles provided supplementation twice daily (22, 31, 33–36), while two articles provided supplementation once and three times daily (21, 32), respectively. The dosage of L-citrulline supplementation varied from 2 grams to 10 grams. Three articles administered interventions for 6 weeks (31, 32, 35), three articles for 4 weeks (22, 34, 36), one article for 2 weeks (33), and one article for 1 week (21).

Regarding outcome measures, eight studies reported PWV data. Among these, five studies provided carotid-femoral PWV (CF-PWV) data (31, 33–36), two studies reported brachial-ankle PWV (BA-PWV) data (21, 32), one study provided carotid-radial PWV (CR-PWV), carotid-distal PWV (CD-PWV), and femoral-ankle PWV (FA-PWV) data (36), and one study did not specify the PWV measurement site (22). Additionally, three studies reported FMD data (22, 34, 36).

### 3.3 Results of meta-analysis

#### 3.3.1 Effects of L-citrulline and watermelon supplementation on pulse wave velocity in middle-aged and elderly individuals

**Figure 2:**
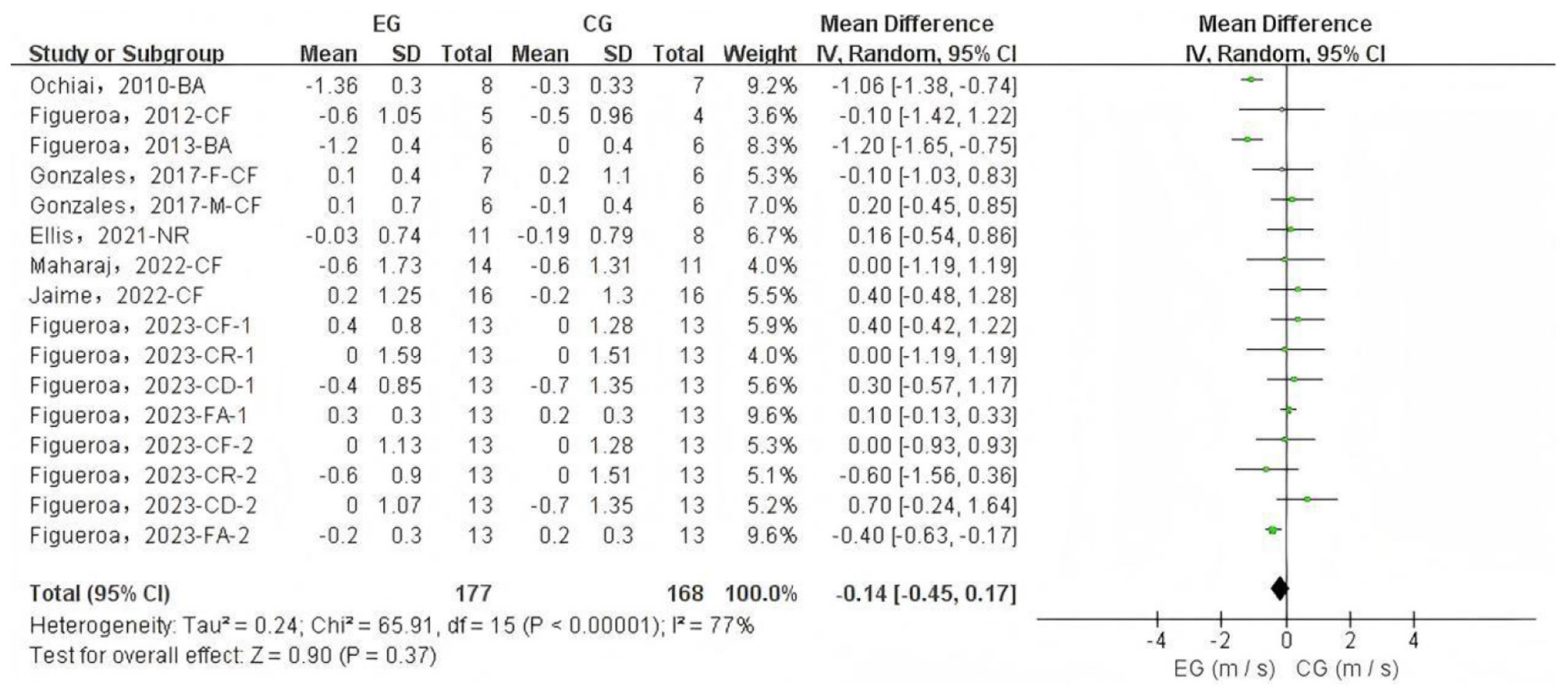
Forest plot of the effects of L-citrulline and watermelon supplementation on pulse wave velocity in middle-aged and elderly adults. CF, carotid-femoral; BA, brachial-ankle; CR, carotid-radial; CD, carotid-distal; FA, femoral-ankle; F, female; M, male; EG, experimental group; CG, control group; M, meter; S, second.

A total of 16 data sets were included in the meta-analysis (Figure 2). The results indicated that L-citrulline and watermelon supplementation did not significantly improve PWV in middle-aged and elderly individuals (WMD = −0.14, 95% CI: −0.45 to 0.17, *p* = 0.37), with substantial heterogeneity observed (*I*^2^ = 77%). Sensitivity analysis, conducted by sequentially excluding individual studies, showed that no single study had a significant impact on the overall results. Additionally, Egger's test indicated no significant publication bias among the included studies (*p* > 0.1).

#### 3.3.2 Effects of L-citrulline and watermelon supplementation on flow-mediated dilation in middle-aged and elderly individuals

**Figure 3:**
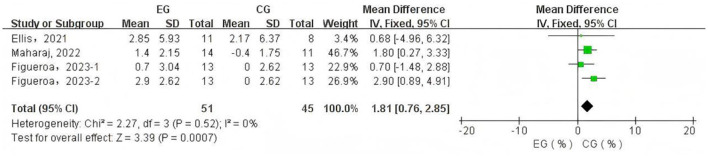
Forest plot of the effects of L-citrulline and watermelon supplementation on flow-mediated dilation in middle-aged and elderly adults. EG, experimental group; CG, control group; %, percentage.

A total of 4 data sets were included in the meta-analysis (Figure 3). The results demonstrated that L-citrulline and watermelon supplementation significantly improved FMD in middle-aged and elderly individuals (WMD = 1.81, 95% CI: 0.76 to 2.85, *p* = 0.0007). Sensitivity analysis, conducted by sequentially excluding individual studies, showed that no single study substantially affected the overall results, indicating robustness. Egger's test indicated no significant publication bias among the included studies (*p* > 0.1).

#### 3.3.3 Subgroup analysis results

**Figure 4:**
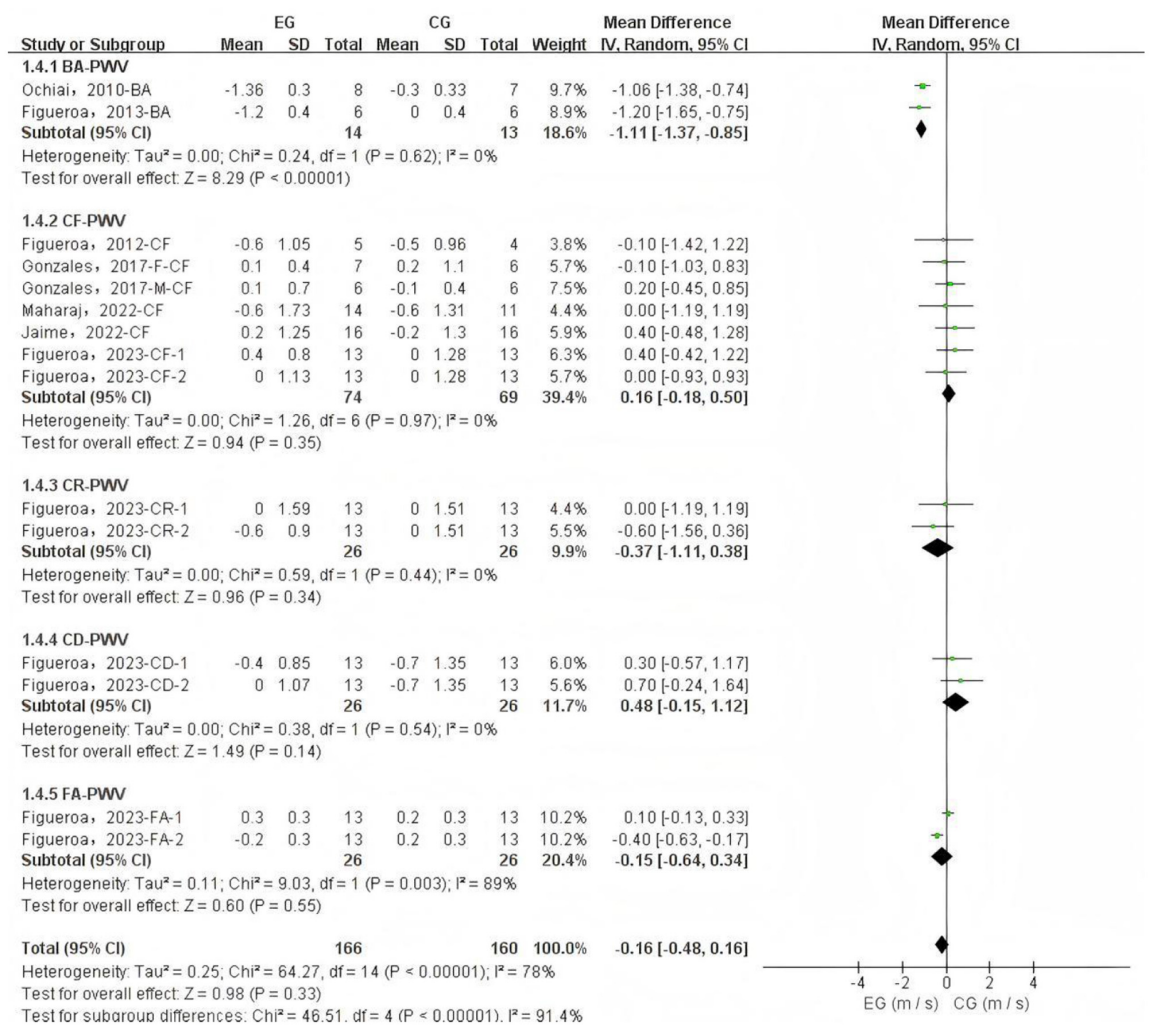
Forest plot of subgroup analysis by pulse wave velocity measurement site. CF, carotid-femoral; BA, brachial-ankle; CR, carotid-radial; CD, carotid-distal; FA, femoral-ankle. F, female; M, male; EG, experimental group; CG, control group; M, meter; S, second.

**Figure 5:**
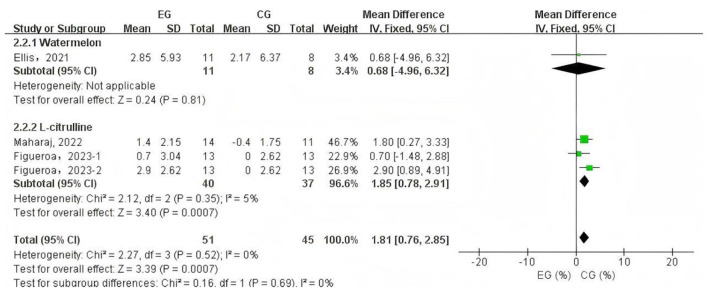
Forest plot of the subgroup analysis of L-citrulline and watermelon effects on flow-mediated dilation. EG, experimental group; CG, control group; %, percentage.

Due to sample size limitations, subgroup analysis was conducted only on PWV data to identify potential influencing factors and sources of heterogeneity. The subgroup analysis based on PWV measurement site (Figure 4) showed that L-citrulline supplementation significantly improved BA-PWV in middle-aged and elderly individuals (WMD = −1.11, 95% CI: −1.37 to −0.85, *p* < 0.00001). The results of other subgroup analyses are summarized in Table 2. **Additionally, based on the subgroup analysis results of the supplementary substances, L-citrulline supplementation significantly improved FMD in middle-aged and elderly individuals (WMD** **=** –**1.85, 95% CI: 0.78 to 2.91**, ***p* =** **0.0007), while watermelon intake showed no improvement in FMD for this population (WMD** **=** **0.68, 95% CI:** –**4.96 to 6.32**, ***p*** **=** **0.81)** (Figure 5).

**Table 2:** Subgroup analysis of potential moderating factors for pulse wave velocity in studies included in the meta-analysis (Random-effects model).

**Group**	**Studies**			**PWV (m/s)**		
	**Number**	**References**	**WMD (95% CI)**	*I* ^2^	***p*** **overall change**	***p*** **for subgroup difference**
**PWV measurement sites**						
BA-PWV	2	(21, 32)	−1.11 (−1.37 to −0.85)	0	< 0.00001	< 0.00001
CF-PWV	5	(31, 33-36)	0.16 (−0.18 to 0.50)	0	0.35	
CR-PWV	1	(36)	−0.37 (−1.11 to 0.38)	0	0.34	
CD-PWV	1	(36)	0.48 (−0.15 to 1.12)	0	0.14	
FA-PWV	1	(36)	−0.15 (−0.64 to 0.34)	89	0.55	
**Supplementary substances**						
L-citrulline	5	(21, 33-36)	−0.01 (−0.48 to 0.45)	80	0.95	0.44
L-citrulline + L-arginine	2	(31, 32)	−0.83 (−1.85 to 0.19)	58	0.11	
L-citrulline + Glutathione	1	(36)	−0.17 (−0.65 to 0.32)	48	0.51	
Watermelon juice	1	(22)	0.16 (−0.54 to 0.86)	/	0.65	
**Daily supplementation frequency**						
2 times	7	(21, 22, 31, 33-36)	−0.01 (−0.35 to −0.24)	72	0.71	< 0.001
3 times	1	(32)	−1.20 (−1.65 to −0.75)	/	< 0.00001	
**Intervention period**						
< 2 weeks	1	(21)	−1.06 (−1.38 to −0.74)	/	< 0.00001	< 0.00001
2-6 weeks	4	(22, 33, 34, 36)	−0.00 (−0.22 to 0.22)	35	1	
≥6 weeks	3	(31, 32, 35)	−0.36 (−1.51 to 0.78)	82	0.53	
**Gender ratio**						
All male	2	(32, 33)	−0.52 (−1.89 to 0.85)	92	0.46	0.56
All female	5	(21, 22, 33, 34, 36)	−0.11 (−0.44 to 0.23)	76	0.53	
Mixed	2	(21, 35)	0.24 (−0.49 to 0.98)	0	0.51	

### 3.4 Risk of bias and quality assessment results

The included literature was assessed as low risk for the following three criteria: randomisation process, missing outcome data, and measurement of the outcome. For deviations from the intended interventions, only one article was rated as having some concerns (32), while the rest were considered low risk. In terms of selection of the reported result, one article was rated as high risk (33), one article as having some concern (22), and the remaining articles were assessed as low risk. Overall, five articles were rated as low risk (21, 31, 34–36), two articles as having some concerns (22, 32), and one article as high risk (33).

The quality assessment of the literature showed that one article scored 4 points (32), one article scored 5 points (21), five articles scored 6 points (22, 31, 33–35), and one article scored 7 points (36). All articles were rated as high-quality literature. Supplementary Material 1 summarizes the specific results of assessing the risk of bias and literature quality.

### 3.5 Publication bias assessment and sensitivity analysis results

**Figure 6:**
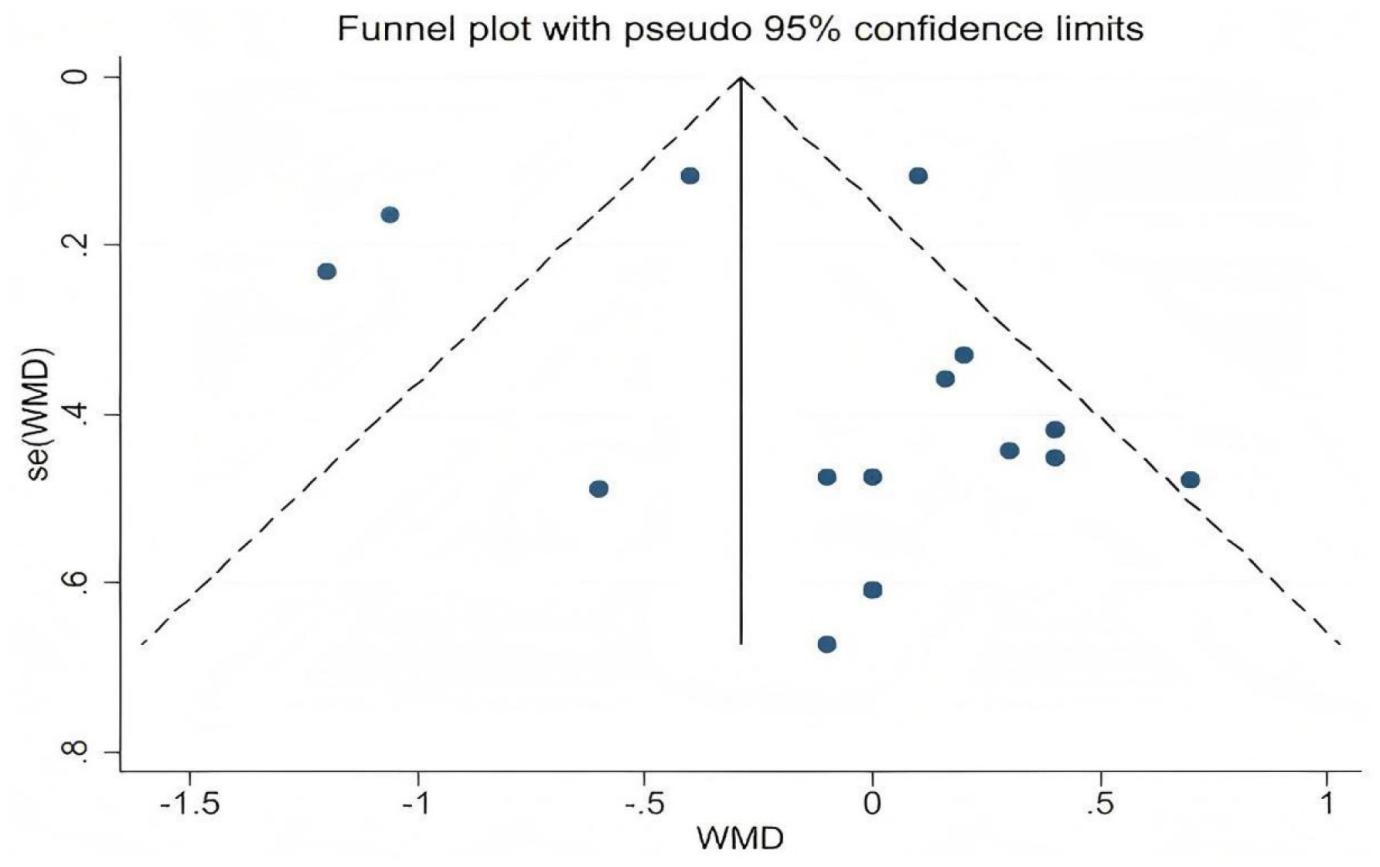
Funnel plot for publication bias assessment of pulse wave velocity.

**Figure 7:**
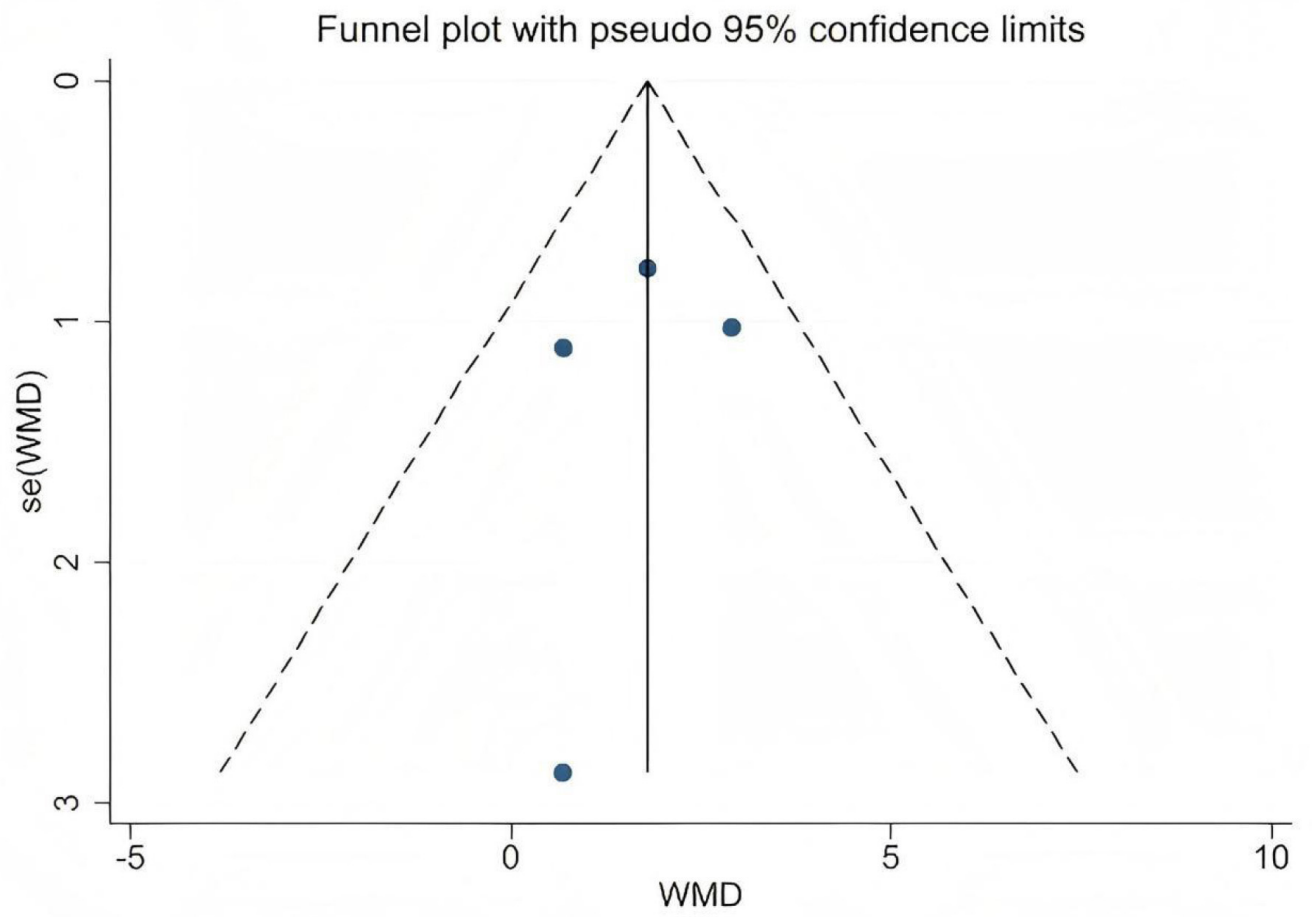
Funnel plot for publication bias assessment of flow-mediated dilation.

Publication bias was visually assessed using funnel plots (Figures 6, 7), and Egger's test indicated no significant publication bias among the included studies (*p* > 0.1). Sensitivity analysis was conducted by sequentially excluding each study to evaluate the stability of the results. The findings showed that no single study had a substantial impact on the overall results, demonstrating the robustness of the PWV and FMD analyses.

## 4 Discussion

The primary aim of this systematic review and meta-analysis is to summarize the research on the effects of L-citrulline supplementation and watermelon intake on AS and endothelial function in older adults. A total of 8 RCTs were included, with an overall sample size of 176 participants. **The meta-analysis results indicate that, compared to placebo, L-citrulline supplementation has a positive impact on vascular function in middle-aged and elderly individuals, significantly improving FMD. However, although PWV did not show improvement, subgroup analysis results still revealed that L-citrulline supplementation significantly reduced BA-PWV. In contrast, watermelon intake did not significantly improve FMD or PWV in middle-aged and elderly individuals**.

### 4.1 Interpretation of study findings

Aging is an inevitable biological process accompanied by progressive vascular dysfunction and degeneration, such as AS (37) and endothelial dysfunction (38), which significantly contribute to the development of CVDs during the aging process (7, 39). With advancing age, PWV values progressively increase (40), reflecting reduced arterial elasticity and increased vascular stiffness; simultaneously, FMD values gradually decrease (41), indicating endothelial dysfunction and impaired vasodilatory capacity. The deterioration of vascular function leads to elevated PWV and reduced FMD, which in turn further contribute to the onset and progression of CVDs (7, 42, 43). This meta-analysis selected FMD, the gold standard for assessing endothelial and vascular function (44, 45), as one of the primary outcome measures to evaluate the effects of L-citrulline and watermelon supplementation on FMD in middle-aged and elderly populations. Endothelial dysfunction induced by aging is a multifaceted pathological process, with core mechanisms involving L-arginine metabolic disorder (46, 47), exacerbated oxidative stress (48, 49), and significant decline in tetrahydrobiopterin (BH_4_) levels (50, 51). In this process, arginase becomes abnormally active (52, 53), hydrolyzing L-arginine to L-ornithine, which not only directly reduces available L-arginine reserves but also competes with eNOS for common substrates (54), further disrupting the normal physiological supply of L-arginine (55). As the aging process progresses, bodily oxidative stress shows a persistent upward trend, particularly with abnormally elevated levels of superoxide and hydrogen peroxide, with the fundamental cause being the gradual enhancement of nicotinamide adenine dinucleotide phosphate (NADPH) oxidase activity, coupled with the progressive decline of antioxidant defense system capabilities (38, 56). Superoxide interacts complexly with NO, generating peroxynitrite (ONOO-), accelerating BH_4_ oxidation, and rapidly converting it to BH_2_, ultimately leading to significant eNOS dysfunction and uncoupling (57, 58). After losing normal function, the eNOS metabolic pathway fundamentally reprograms, with products shifting from NO to large amounts of superoxide and ONOO-, severely deviating from normal physiological function (59). The upregulation of arginase activity and oxidative stress-induced eNOS uncoupling interact, forming a vicious cycle that synergistically reduces NO} bioavailability and ultimately impairs }the endothelium-dependent vasodilation regulatory mechanism (55). The resulting NO level reduction fails to effectively activate the Guanylate Cyclase (GC)–Guanosine-5′-Triphosphate (GTP)–Cyclic Guanosine Monophosphate (cGMP) signaling pathway, causing impaired vasodilation function (60). Simultaneously, ornithine promotes polyamine and proline generation, thereby inducing collagen synthesis and cell proliferation, which promoting AS (55). Our research findings indicate that L-citrulline supplementation positively contributed to the improvement of endothelial cell function in middle-aged and elderly individuals, specifically leading to a significant enhancement in FMD. The primary mechanism of L-citrulline supplementation improving FMD is its ability to increase L-arginine supply to promote NO generation. NO, as a gaseous molecule released from the endothelium, initiates a series of signal cascade reactions, activating the GC-GTP-cGMP pathway to increase cGMP levels as a second messenger (60). Under NO action, this promotes smooth muscle cell relaxation in conduit and resistance arteries, facilitating vasodilation (60). Our research results indicate that L-citrulline supplementation play a positive role in improving endothelial cell function in middle-aged and elderly individuals, specifically resulting in significant improvement in FMD (Figure 5). Research shows that when FMD increases by 1%, the risk of future cardiovascular events decreases by 12% (43). Our results highlight the potential benefits of L-citrulline supplementation in improving endothelial cell function and preventing CVDs. The primary mechanism of L-citrulline supplementation improving FMD is its ability to increase L-arginine supply to promote NO generation. NO, as a gaseous molecule released from the endothelium, initiates a series of signal cascade reactions, activating the GC-GTP-cGMP pathway to increase cGMP levels as a second messenger (60). Under NO action, this promotes smooth muscle cell relaxation in conduit and resistance arteries, facilitating vasodilation (60). Although the meta-analysis results show that L-citrulline and watermelon significantly improve FMD overall (Figure 3), the experimental studies included in this article showed inconsistent results. Ellis et al. found that a four-week supplementation of 100% watermelon juice in healthy postmenopausal women did not significantly improve FMD in that population (22). Meanwhile, Figueroa et al.'s study on healthy postmenopausal women conducted a four-week supplementation of L-citrulline and L-citrulline combined with glutathione, revealing that both supplements significantly improved FMD, but only the group receiving L-citrulline combined with glutathione reached statistical significance (36). This indicates that watermelon juice and L-citrulline alone have limited effects on improving FMD in healthy postmenopausal women. However, a four-week supplementation of L-citrulline by Maharaj et al. in hypertensive postmenopausal women showed that L-citrulline significantly improved FMD in that population (34). Based on the existing evidence, we can infer that watermelon juice supplementation seems to have failed to improve FMD. Secondly, the improvement effect of L-citrulline on FMD may be related to the participants' health status. Moreover, the supplementation of L-citrulline combined with glutathione may be superior to the individual watermelon juice and L-citrulline supplementation. In summary, although L-citrulline and watermelon supplementation significantly improved FMD overall, the above results should be interpreted with caution due to sample size limitations (Figure 3). For middle-aged and elderly individuals, there is also controversy regarding the improvement of PWV by L-citrulline and watermelon supplementation. L-citrulline and watermelon supplementation did not significantly improve PWV (with no significant improvement in CF-PWV, CR-PWV, CD-PWV, and FA-PWV), but BA-PWV was significantly reduced by 1.11 m/s. First of all, the limited number of studies included in this article and the small sample sizes may have influenced this result. Secondly, although L-citrulline and watermelon supplementation can improve endothelial cell function through NO-mediated mechanisms (12, 61, 62), aging-induced AS involves various complex mechanisms, such as extracellular matrix stiffness due to collagen, changes in vascular smooth muscle cell characteristics, inflammation, and endothelial dysfunction (63–65), all of which are regulated by multiple factors, with mechanisms not fully elucidated. Therefore, the improvement in endothelial function due to L-citrulline and watermelon supplementation is only part of the mechanism for AS improvement. Existing evidence indicates that L-citrulline supplementation can improve levels of pro-inflammatory factors, lipid profiles, and biomarkers, such as promoting a decrease in tumor necrosis factor-α (66), interleukin-6 (67), high-sensitivity C-reactive protein (66–68), glycated hemoglobin (66), and low-density lipoprotein (68), while increasing high-density lipoprotein levels (66). Supplementation with L-citrulline may enhance the reutilization of the arginine cycle, thereby affecting the levels of these biomarkers and exhibiting notable anti-inflammatory properties. Specifically, L-citrulline may reduce the expression of the Toll-like receptor 4 (TLR4) gene, which subsequently inhibits the activation of nuclear factor kappa B (NF-κB) and the production of tumor necrosis factor-α (69–71). Moreover, L-citrulline may further alleviate oxidative stress by enhancing the activity of superoxide dismutase (SOD) (72), thus contributing to the suppression of inflammatory responses. Improvements in these biomarkers are significant for reducing AS (64, 65, 73–75). Although existing evidence shows that L-citrulline and watermelon supplementation can improve endothelial function and some biomarker levels, our meta-analysis results indicate that L-citrulline and watermelon supplementation did not significantly improve PWV in middle-aged and elderly individuals overall. Clinically, CF-PWV and BA-PWV are commonly used indicators for measuring AS, effectively reflecting AS status and predicting overall cardiovascular events (76, 77). However, our subgroup analysis only showed significant improvement in BA-PWV. The lack of significant improvement in PWV, especially in CF-PWV, may also be due to a relatively short intervention duration. The progression of AS is a complex and long-term process (65), and the longest intervention time in the studies included in this article was six weeks; a shorter intervention period may limit the benefits of L-citrulline supplementation and watermelon intake on AS. Therefore, future research needs to design longer intervention periods, increase sample sizes, and conduct more longitudinal comparative clinical trials to further validate the effects of L-citrulline and watermelon supplementation on PWV.

### 4.2 Limitations

This study has some unavoidable limitations. First, there are relatively few studies included. Therefore, we included studies that combine L-citrulline with other supplementary substances, which may have some impact on the results. Second, the small sample size also limited the subgroup analyses. Many subgroup results need to be interpreted with caution due to insufficient sample sizes, and some even could not undergo subgroup analysis. Third, among the included studies, some utilized an intervention method combining L-citrulline with other supplementary substances. Despite our relevant subgroup analyses, this still had a certain impact on the overall results. Finally, the intervention periods in the included studies were generally short, which may limit the effectiveness of improvements in the analyzed biomarkers.

### 4.3 Clinical practice recommendations

Based on the results of this systematic review and meta-analysis, the design of future related RCTs should focus on the following aspects. First, the longest intervention period in the studies included in this article was only 6 weeks, and the short intervention time may have limited the observation of significant improvements in PWV. An RCT without a placebo group indicated that there was a significant decrease in FA-PWV and BA-PWV after 8 weeks of L-citrulline supplementation in postmenopausal women (78). However, a 4-week intervention RCT included in this article did not observe a significant improvement in FA-PWV (36). Therefore, the differences in intervention duration and study design may impact the results. Future large-sample, long-term follow-up RCTs should be conducted to fully assess the long-term effects of L-citrulline and watermelon supplementation on PWV. Secondly, more detailed longitudinal comparative studies should be designed to explore the mechanisms behind functional changes in different vascular sites (such as central and peripheral arteries), particularly the differences in responses between BA-PWV and CF-PWV, to reveal their potential physiological and pathological mechanisms. Third, some evidence suggests that L-citrulline combined with other interventions (such as exercise or other supplementary substances) may exhibit a synergistic effect in improving vascular function, providing new research ideas and directions for enhancing cardiovascular health in the elderly population. Finally, future studies need to strictly control and clarify key variables such as the types, doses, and frequencies of auxiliary supplements to systematically evaluate their specific effects on improving vascular function, thereby increasing the accuracy and reproducibility of the research results. By making these improvements, it is hoped to provide a solid scientific basis for the clinical application of L-citrulline and watermelon supplementation in the vascular health management of middle-aged and elderly individuals.

## 5 Conclusion

This systematic review and meta-analysis indicate that L-citrulline supplementation has an overall positive effect on vascular health in middle-aged and older adults. This is primarily reflected in the significant reduction of BA-PWV and the marked improvement in FMD associated with L-citrulline supplementation. However, watermelon intake did not significantly improve FMD and PWV in this population. Future large-scale studies are needed to confirm the effects of watermelon on vascular function.

## Data Availability

The original contributions presented in the study are included in the article/Supplementary material, further inquiries can be directed to the corresponding author/s.
