# Supplementary material for: Effects of L-citrulline supplementation and watermelon intake on arterial stiffness and endothelial function in middle-aged and older adults: a systematic review and meta-analysis of randomized controlled trials
Source: Front Nutr. 2025 Nov 13;12:1632952. doi: 10.3389/fnut.2025.1632952 (PMC12658987; doi:10.3389/fnut.2025.1632952)

**Table 1. Risk of Bias Assessment using RoB 2**

| **Study lD** | **Randomisation process** | **Deviations from the intended interventions** | **Mising outcome data** | **Measurement of the outcome** | **Selection of the reported result** | **Overall risk-of-bias judgment** |
| --- | --- | --- | --- | --- | --- | --- |
| Ochiai, 2010 | Low risk | Low risk | Low risk | Low risk | Low risk | Low risk |
| Figueroa, 2012 | Low risk | Low risk | Low risk | Low risk | Low risk | Low risk |
| Figueroa, 2013 | Low risk | Some concerns | Low risk | Low risk | Low risk | Some concerns |
| Gonzales，2017 | Low risk | Low risk | Low risk | Low risk | High risk | High risk |
| Ellis, 2021 | Low risk | Low risk | Low risk | Low risk | Some concerns | Some concerns |
| Maharaj, 2022 | Low risk | Low risk | Low risk | Low risk | Low risk | Low risk |
| Jaime，2022 | Low risk | Low risk | Low risk | Low risk | Low risk | Low risk |
| Figuero, 2023 | Low risk | Low risk | Low risk | Low risk | Low risk | Low risk |

**Table 2. literature quality assessment**

| **Study lD** | **Random sequence production** | **Allocation concealment** | **Blinding method** | **Withdrawal** | **Total score of Jadad scale** |
| --- | --- | --- | --- | --- | --- |
| Ochiai, 2010 | 2 | 1 | 2 | 0 | 5 |
| Figueroa, 2012 | 2 | 1 | 2 | 1 | 6 |
| Figueroa, 2013 | 2 | 1 | 1 | 0 | 4 |
| Gonzales，2017 | 2 | 1 | 2 | 1 | 6 |
| Ellis, 2021 | 2 | 1 | 2 | 1 | 6 |
| Maharaj, 2022 | 2 | 1 | 2 | 1 | 6 |
| Jaime，2022 | 2 | 1 | 2 | 1 | 6 |
| Figuero, 2023 | 2 | 2 | 2 | 1 | 7 |

**Search strategy:**

| **1.PubMed: 10** |
| --- |
| Set1:("Citrulline"[Mesh]) OR ((citrulline malate[Title/Abstract]) OR (l-citrulline[Title/Abstract])) |
| Set2:("Citrullus"[Mesh]) OR ((watermelon[Title/Abstract]) OR (citrullus lanatus[Title/Abstract])) |
| Set3:("Vascular Stiffness"[Mesh]) OR ((((arterial stiffness[Title/Abstract]) OR (aortic stiffness[Title/Abstract])) OR (pulse wave velocity[Title/Abstract])) OR (PWV[Title/Abstract])) |
| Set4:((((((((((((endothelial function[Title/Abstract]) OR (endothelium[Title/Abstract])) OR (vascular endothelium[Title/Abstract])) OR (vascular reactivity[Title/Abstract])) OR (vascular[Title/Abstract])) OR (vasodilation[Title/Abstract])) OR (brachial artery[Title/Abstract])) OR (brachial artery dilation[Title/Abstract])) OR (flow mediated dilation[Title/Abstract])) OR (artery blood flow[Title/Abstract])) OR (artery dilation[Title/Abstract])) OR (flow mediated[Title/Abstract])) OR (FMD[Title/Abstract]) |
| Set5: ((random[Title/Abstract]) OR (randomized controlled trial[Title/Abstract])) OR (RCT[Title/Abstract]) |
| Set6: #1 OR #2 |
| Set7: #3 OR #4 |
| Set8: #5 AND #6 AND #7 |
|  |
| **2.Cochrane：138** |
| Set1:MeSH descriptor: [Citrulline] in all MeSH products |
| Set2:MeSH descriptor: [Citrullus] explode all trees |
| Set3:MeSH descriptor: [Vascular Stiffness] explode all trees |
| Set4:(citrulline malate):ti,ab,kw or (l-citrulline) |
| Set5:(watermelon):ti,ab,kw or (citrullus lanatus) |
| Set6:(arterial stiffness):ti,ab,kw or (aortic stiffness):ti,ab,kw or (pulse wave velocity):ti,ab,kw or (PWV) |
| Set7:(endothelial function):ti,ab,kw or (endothelium):ti,ab,kw or (vascular endothelium):ti,ab,kw or (vascular reactivity)i,ab,kw or (vascular):ti,ab,kw or (vasodilation)i,ab,kw or (brachial artery):ti,ab,kw or (brachial artery dilation)i,ab,kw or (flow mediated dilation):ti,ab,kw or (artery blood flow):ti,ab,kw or (artery dilation):ti,ab,kw or (flow mediated):ti,ab,kw or (FMD) |
| Set8:#1 OR #4 |
| Set9:#2 OR #5 |
| Set10:#8 OR #9 |
| Set11:#3 OR #6 OR #7 |
| Set12:#10 AND #11 |
|  |
| **3.Embase：18** |
| Set1:'citrulline'/exp |
| Set2:'citrullus'/exp |
| Set3:'arterial stiffness'/exp |
| Set4:'citrulline malate':ab,ti OR 'l-citrulline' |
| Set5:'watermelon':ab,ti OR 'citrullus lanatus' |
| Set6:'vascular stiffness':ab,ti OR 'aortic stiffness':ab,ti OR 'pulse wave velocity':ab,ti OR 'pwv' |
| Set7:'endothelial function':ab,ti OR 'endothelium':ab,ti OR 'vascular endothelium':ab,ti OR 'vascular reactivity':ab,ti OR 'vascular':ab,ti OR 'vasodilation':ab,ti OR 'brachial artery':ab,ti OR 'brachial artery dilation':ab,ti OR 'flow mediated dilation':ab,ti OR 'artery blood flow':ab,ti OR 'artery dilation':ab,ti OR 'flow mediated':ab,ti OR 'fmd' |
| Set8:'random':ab,ti OR 'randomized controlled trial':ab,ti OR 'rct' |
| Set9:#1 OR #2 OR #4 OR #5 |
| Set10:#3 OR #6 OR #7 |
| Set11:#8 AND #9 AND #10 |
|  |
| **4.Web of science：242** |
| Set1:((TS=(citrulline)) OR TS=(citrulline malate)) OR TS=(l-citrulline) |
| Set2:((TS=(citrullus)) OR TS=(watermelon)) OR TS=(citrullus lanatus) |
| Set3:((((TS=(vascular stiffness)) OR TS=(arterial stiffness)) OR TS=(aortic stiffness)) OR TS=(pulse wave velocity)) OR TS=(PWV) |
| Set4:((((((((((((TS=(endothelial functio)) OR TS=(endothelium)) OR TS=(vascular endothelium)) OR TS=(vascular reactivity)) OR TS=(vascular)) OR TS=(vasodilation)) OR TS=(brachial artery)) OR TS=(brachial artery dilation)) OR TS=(flow mediated dilation)) OR TS=(artery blood flow)) OR TS=(artery dilation)) OR TS=(flow mediated)) OR TS=(FMD) |
| Set5:((TS=(random)) OR TS=(randomized controlled trial)) OR TS=(RCT) |
| Set6:#1 OR #2 |
| Set7:#3 OR 4 |
| Set8:#5 AND #6 AND #7 |


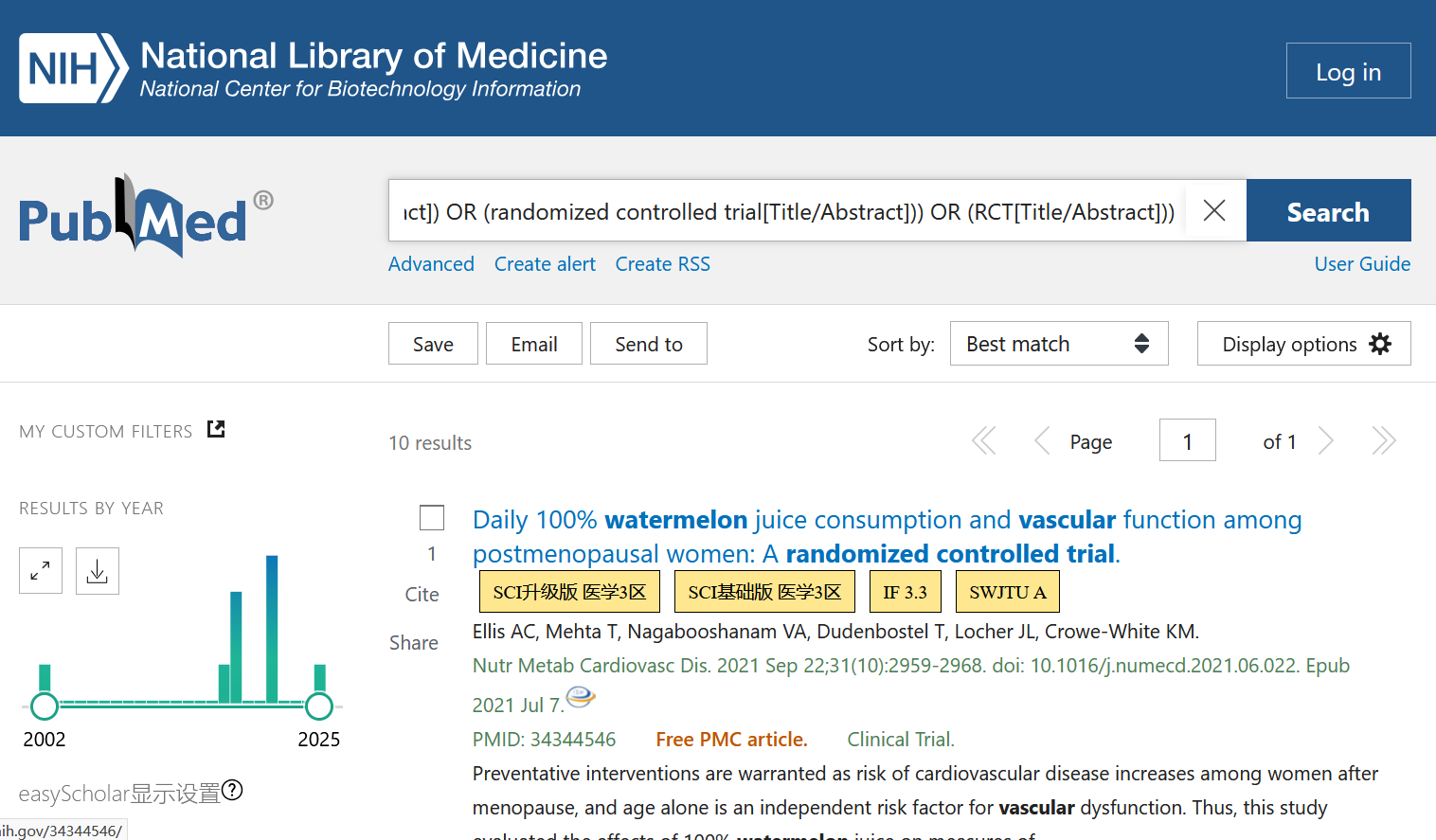

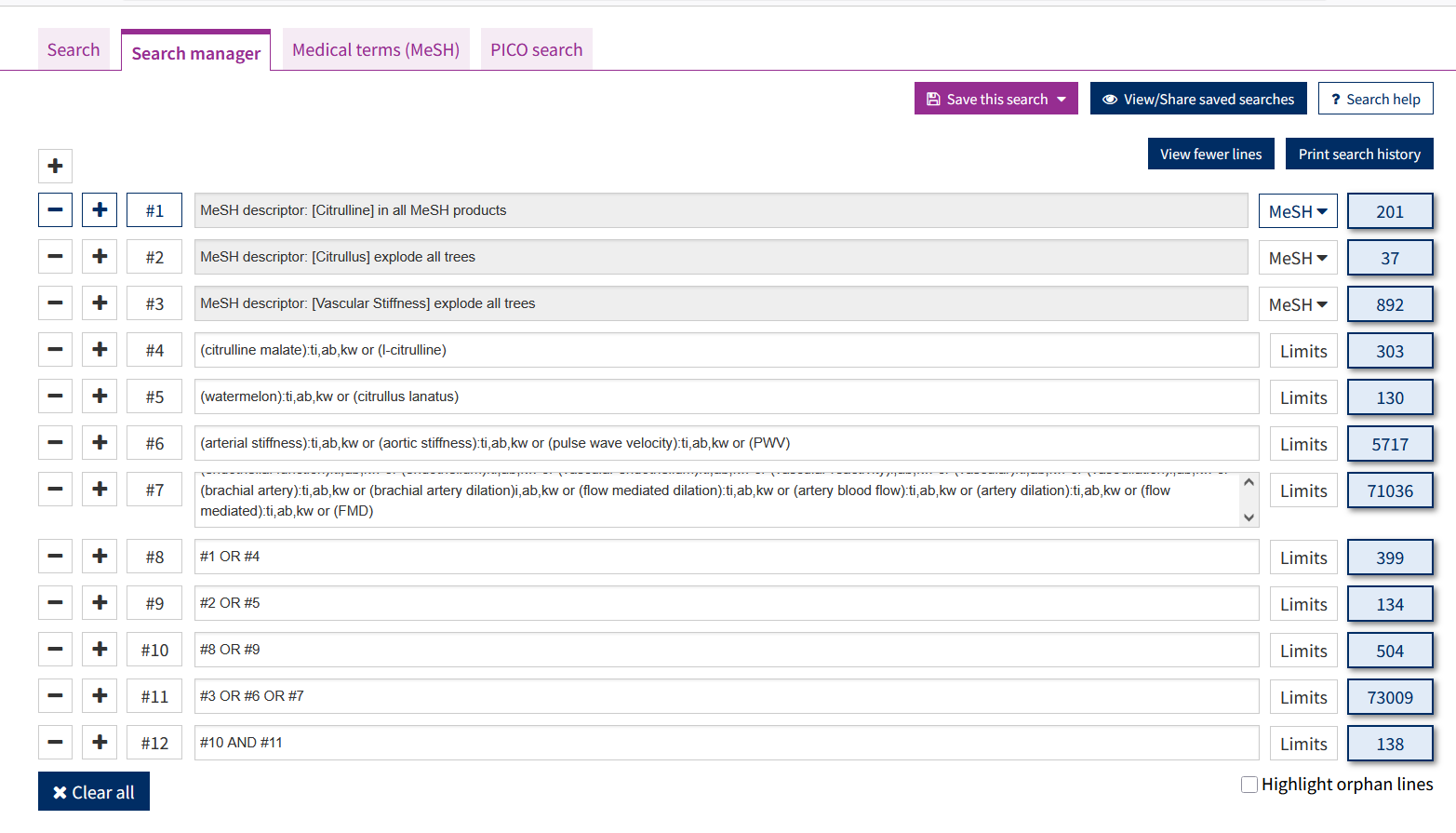


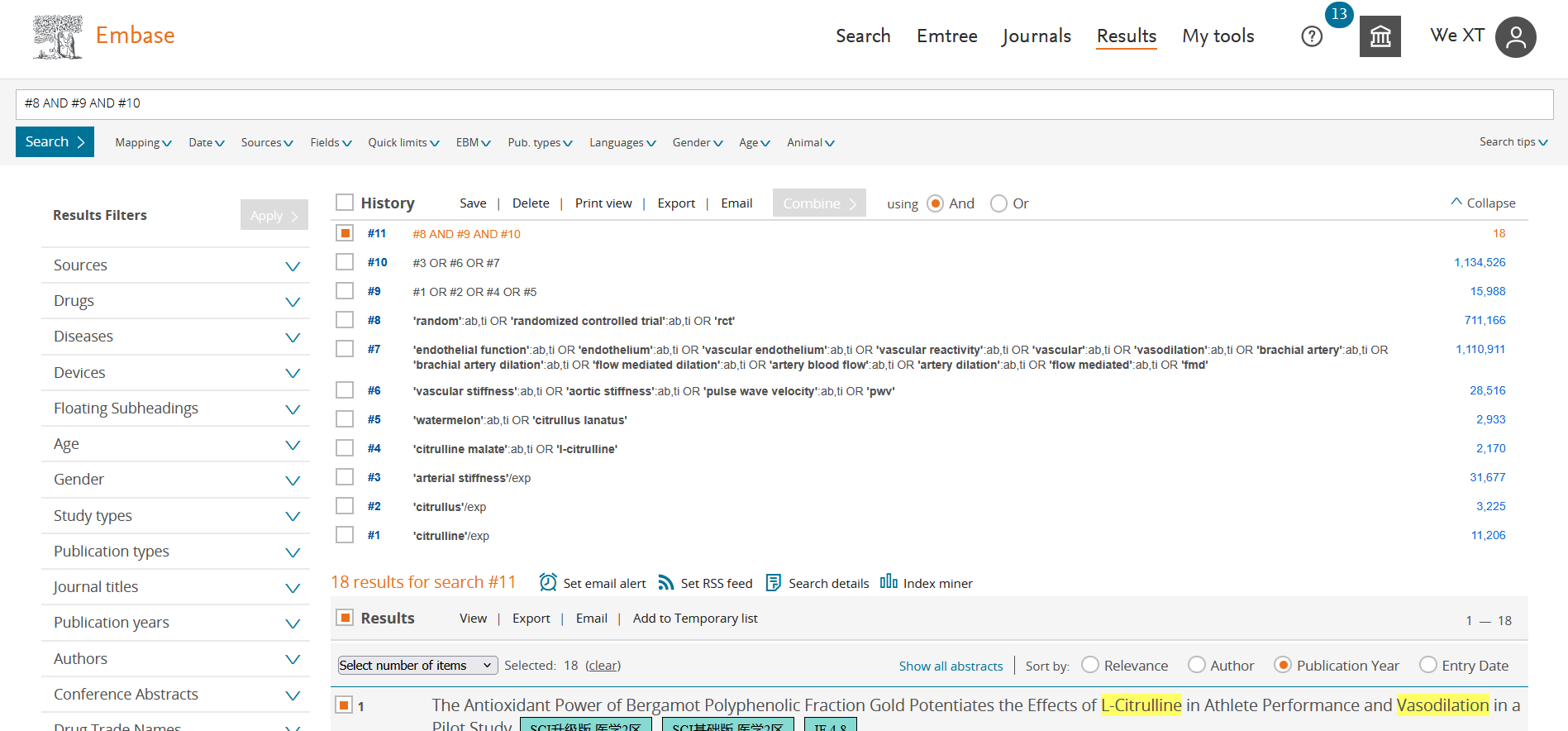


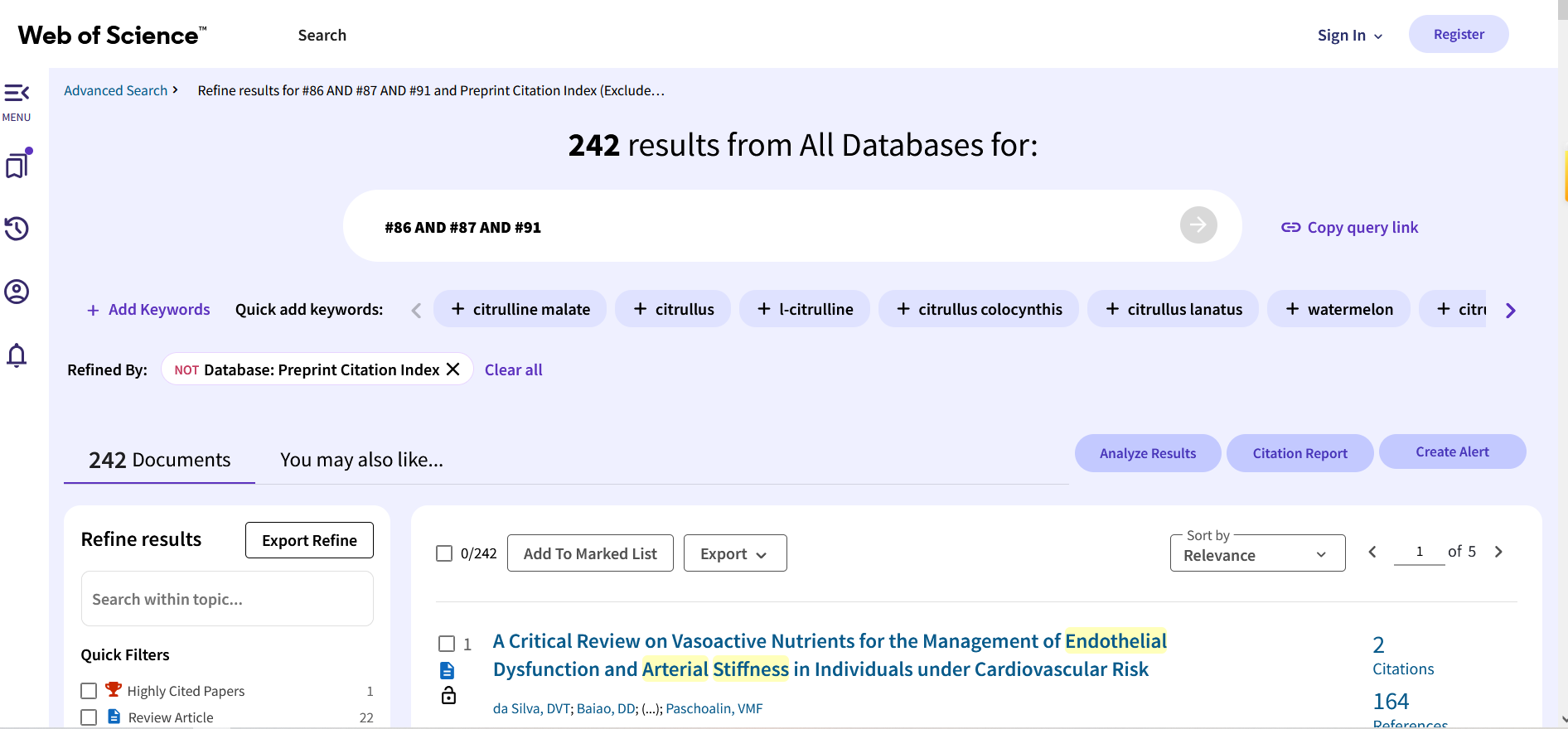

Supplement: Supplementary file 1 [file Table_1.docx]
